# Individual Contrast Preferences in Natural Images

**DOI:** 10.3390/jimaging10010025

**Published:** 2024-01-18

**Authors:** Olga Cherepkova, Seyed Ali Amirshahi, Marius Pedersen

**Affiliations:** Department of Computer Science, Norwegian University of Science and Technology, 2802 Gjøvik, Norway; s.ali.amirshahi@ntnu.no (S.A.A.); marius.pedersen@ntnu.no (M.P.)

**Keywords:** perception, individual preferences, contrast, 3AFC

## Abstract

This paper is an investigation in the field of personalized image quality assessment with the focus of studying individual contrast preferences for natural images. To achieve this objective, we conducted an in-lab experiment with 22 observers who assessed 499 natural images and collected their contrast level preferences. We used a three-alternative forced choice comparison approach coupled with a modified adaptive staircase algorithm to dynamically adjust the contrast for each new triplet. Through cluster analysis, we clustered observers into three groups based on their preferred contrast ranges: low contrast, natural contrast, and high contrast. This finding demonstrates the existence of individual variations in contrast preferences among observers. To facilitate further research in the field of personalized image quality assessment, we have created a database containing 10,978 original contrast level values preferred by observers, which is publicly available online.

## 1. Introduction

Personalized approaches are gaining increasing popularity not only in the field of Image Quality Assessment (IQA) [1] but also in various other domains [2]. For years, subjective experiments have delivered results in the form of Mean Opinion Scores (MOSs). An MOS is defined by the International Telecommunication Union as a measurement of the voice quality of an interaction: the mean of individual “values on a predefined scale that a subject assigns to his opinion of the performance of a system quality” [3]. There exist recommendations and guidelines for several aspects related to subjective experiments, such as the number of observers [4,5], the number of stimuli [6], the type of stimuli [6], the size of the experiment [5], outlier removal [5], etc. Currently, most of the largest databases in the field of IQA, such as [7,8,9], only provide MOSs. This is mainly due to their convenience for use in quality prediction and image enhancement. Predicting quality based on MOS values is cost-effective and time-efficient and thus fulfills the needs of most observers: that is, if their opinion follows a normal distribution around the mean. However, considering different distributions, for example binomial or homogeneous, will lead to weak correlation with the MOS and an inaccurate representation of observer opinions.

Natural variation in human perception is an established phenomenon characterized by unique individual variations. Variances extend beyond color blindness, visual acuity, and pathological conditions to include variations in perceptual experiences of healthy individuals [10]. Contrast sensitivity is also different for individuals and changes with age [11]. “The state of the observer plays a fundamental role in determining the things he sees” [12] and depends on many biological and psychological factors. Although the existence of these differences in addition to personal preferences is widely acknowledged, for the sake of simplicity, it is often overlooked in practical applications such as image quality assessment. An MOS not only serves as a representation of all scores given to the same image in subjective quality experiments but is also used when evaluating the performance of different objective Image Quality Metrics (IQMs). The accuracy of different IQMs is often calculated in accordance with the correlation it provides with the MOS and leaves aside individual differences. Therefore, we are taking a step forward in another direction by proposing the use of a more complex, but yet more accurate, personalized IQA. Although personalized IQAs are a vast field in themselves, our initial focus is to analyze individual differences in one of the influencing factors in image quality [13]: the contrast image attribute.

In this work, we focus on contrast, a specific image attribute that not only influences the perception of image quality [14] but also causes one of the largest variabilities among observers when used as a type of distortion in testing image quality preferences [1]. Exploring this particular attribute, our objective is to identify distinctive observer preferences in order to use this information to improve personalized image quality in the future. Our aim is to uncover potential groups with similar preferences in the level of contrast in the image. Unfortunately, major databases used in IQA only provide MOSs, leading to the inability to test existing and develop new IQMs customized to personalized preferences. Therefore, there exists a need for a dataset that provides individual preference values for contrast. To accomplish this, we collected a dataset containing contrast preferences given by 22 observers for 499 natural landscape images.

This paper is organized as follows. Section 2 presents an overview of personalized IQA methods and findings in the literature. Section 3 provides details about the database collection and subjective experiment procedure. The results are provided in Section 4, followed by a conclusion of the work and possible future directions in Section 5.

## 2. Background

In this paper, we evaluate individual preferences at different contrast levels for a wide range of images that all can be classified as images taken from natural landscapes (Table 1). This attribute was chosen among others, such as lightness, sharpness, colorfulness, etc., because it leads to one of the largest variabilities between observer opinions when evaluating image quality [1]. Cherepkova et al. [15] show that observers have distinguishable patterns with regard to contrast distortion when judging image quality, where some observers rate images with higher contrast to have higher quality and vice versa. Contrast is a fundamental image attribute that can influence how the image is perceived by observers and has a significant impact on IQA [16] and enhancement [17]. The effect of contrast on perceived image quality was discovered in early works; an example of such a subjective experiment was reported in the work of Roufs and Goossens in 1988 [13] and by Roufs et al. in 1994 [18]. Contrast as a feature is used in different no-reference IQMs that predict perceived image quality: for example, in the IQM based on global statistical features proposed by Varga [19] or the histogram equalization algorithm proposed by Chen [20]. Other metrics have been developed based on contrast as a distortion type: for example, the no-reference IQMs presented by Ziaei Nafchi [21], Liu [22], and Fang [23].

Contrast is often measured using different mathematical measures and metrics [14], such as the Michelson contrast [24], RMS contrast, Weber contrast [25], Local contrast [26], and contrast-to-noise ratio [27]. However, these metrics are less susceptible to individual preferences as they are based on objective measures of image contrast rather than subjective perception.

Personal preferences have also been explored in studies that have investigated relationships between different image attributes. For example, Calabria and Fairchild [28] studied the relationship between perceived contrast and observer preferences using a paired comparison of five pictorial images. They also investigated the role of other image attributes, such as lightness, chroma, and sharpness, in determining the perceived image contrast. They found a non-linear relationship between perceived contrast and preferred images that resulted in a reversed U-shaped form and highlighted an optimal point of preferred contrast for each type of content. They developed a model that, independent of image content, predicts the preferred image contrast. Kadyrova et al. [29] carried out a subjective experiment wherein a group of users adjusted images according to sharpness, saturation, warmth, brightness, and contrast. The adjustments changed between observers, which indicated individual differences. The contrast attribute had a large difference in the adjustments made by the users. These images were further evaluated by observers and showed that content plays a role in what was preferred by observers.

**Table 1:** Examples of images in the database with different degrees of RMS contrast, busyness [30], colorfulness [31], lightness [32], and complexity [32] with their corresponding values presented below each image.

	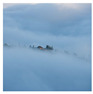	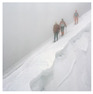	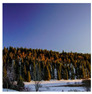	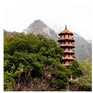	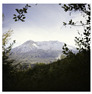
RMS Contrast	0.05	0.06	0.21	0.35	0.37
	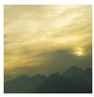	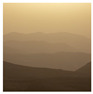	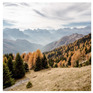	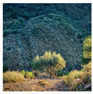	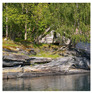
Busyness	0.08	0.16	51.27	99.93	99.95
	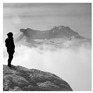	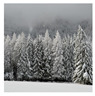	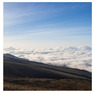	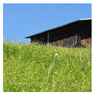	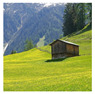
Colorfulness	0.008	2.1	18.04	45.14	45.45
	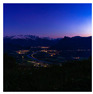	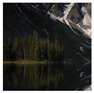	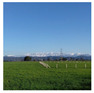	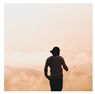	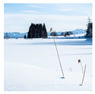
Lightness	4.83	8.06	52.8	90.11	93.4
	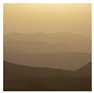	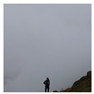	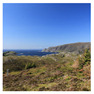	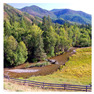	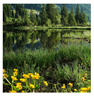
Complexity	0.06	0.07	1.15	3.84	3.88

Individual preferences in general have been studied more in the area of aesthetics [33,34]. Such studies explore the influence of aesthetic-related, high-level, or describable visual image characteristics (the rule of thirds, golden ratio, visual balance, composition, illumination, etc.) [35,36,37], use of personality traits and individual characteristics [38,39,40], emotions [41], and a combination of image- and individual-related information [42,43] to build a personalized aesthetics assessment model. Other works use machine learning [44] and deep learning [2,40,45,46,47] techniques to predict individual preferences based on the features of the image and the viewer him/herself.

Individual preferences are also used to adjust image parameters and enhance images to provide a better experience for each viewer. Kim et al. [48] recorded user preferences in preference vectors, which were then used to train a deep learning model that changes new image attributes according to user preferences collected from other images. A similar two-stage approach was used by Kang et al. [49] and Bianco et al. [50], whereby they employed a training set of images for users to evaluate and a machine learning algorithm to learn the individual’s preferences and adjust the new images accordingly. Caicedo et al. [51] added another stage, where users give collaborative feedback to the enhanced images based on their personal preferences. These ratings are then used to learn a personalized enhancement operator for each user with a collaborative filtering approach. These studies demonstrate the potential of the use of individual preferences to advance the media quality industry.

Differences in perception between observers have been assessed in other areas. Gigilashvili et al. [52] reported variability in glossiness perception between participants after conducting a series of psychophysical experiments. A group-based analysis of the data was then used to find similar behaviors between observers to assess glossiness. Engelke et al. [53] focused on observer differences in multimedia quality assessment and proposed a framework for inter-observer variability analysis. Zhang et al. [54] investigated the preferred level of sharpness for different image content using a rank-order test.

In general, these studies work toward developing methods that account for individual preferences in various domains. This study focuses on identifying individual preferences for a single image attribute and grouping observers with similar preferences.

## 3. Experiment

In this section, we present an overview of our subjective experiment, which was conducted in a controlled laboratory environment. We provide detailed information on the dataset used, the design of the experiment, and the procedure. This includes information on how the images were presented to the participants, the methodology for selecting the initial contrast, the process of adjusting the contrast, and the criteria employed as the stopping rule.

### 3.1. Dataset Preparation

To create the dataset for this work, we used images from the Pixabay website [55], which are distributed under the Simplified Pixabay License allowing non-commercial use without attribution. The minimum resolution of the downloaded images was 1920 × 1080 pixels. The images were then resized and cropped to 600 × 600 pixels to ensure that three images could fit the width of the full HD-resolution screen used in our experiments without further rescaling. The images were cropped to ensure that they featured a main object or a simple scene, and we avoided images with many details that could distract observers and cause their attention to be divided. This was done to avoid variability in the evaluation results due to different points of attention while judging the contrast of different objects within an image. The frequency-tuned salient region detection method [56] was used to identify the salient region of the image and crop around it. Subsequently, the results were manually verified and, if necessary, the region was adjusted. This approach aimed to direct the focus of the observers to similar region(s) or object(s) while minimizing saliency-based variability as much as possible. It is important to note that this factor inevitably influences the judgment to some degree, but the effect is tolerable compared to using complex scenes.

To avoid any bias that may arise from the content of the images, 499 images with the tag “mountain” were randomly selected from thousands of downloaded images. This was done to ensure that the images used in the experiment are representative of a wide variety of images taken from nature while at the same time minimizing the influence of content variability on the research results. However, the images still contained buildings, memory colors such as green grass or blue sky, snow, day and night images, sunsets, and sunrises.

**Figure 1:**
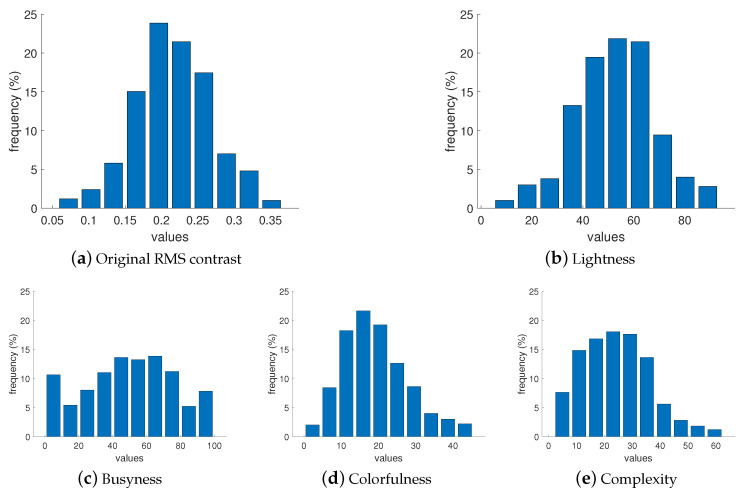
Characteristics of the attributes for images in our dataset.

**Figure 2:**
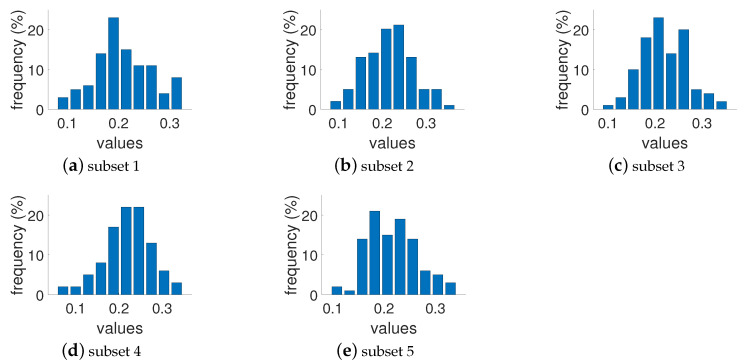
Original RMS contrast distribution in each of the five subsets.

**Figure 3:**
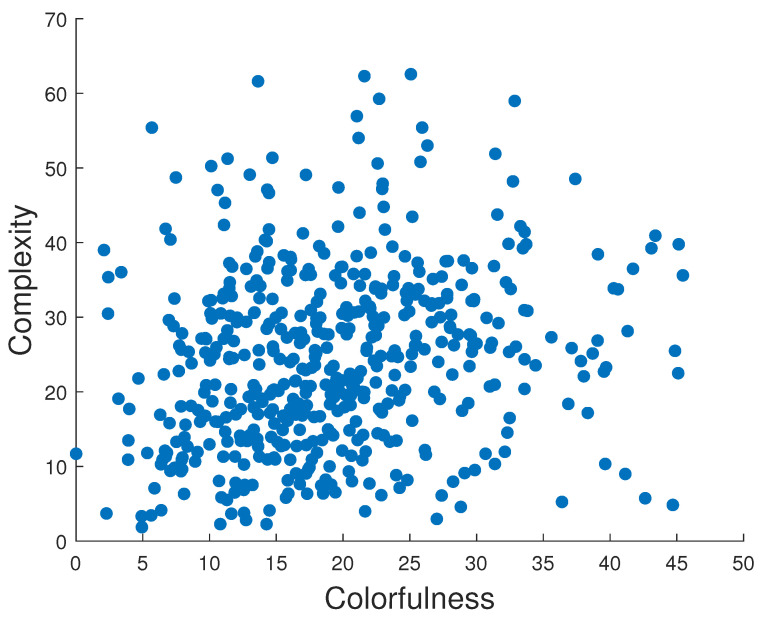
Colorfulness vs. complexity of the images in our dataset.

The selected images were then analyzed to ensure that the dataset used in the experiment represented a diverse range of image attributes and was suitable for evaluation at different levels of these attributes. The distribution of selected image attributes is presented in Figure 1. The presented attributes include the original contrast values, which were calculated according to RMS contrast formula and that demonstrate the extent to which pixels deviate from the mean luminance; this is calculated as

(1) $$\mathrm{RMS}\;\mathrm{Contrast}=\sqrt{\frac{1}{MN}\sum_{i=0}^{N-1}\sum_{j=0}^{M-1}{\left(I_{ij}-\bar{I}\right)}^{2}},$$

where $I_{ij}$: intensity of $i_{th}$ and $j_{th}$ pixel, $\bar{I}$: average intensity of all pixels, *M* and *N*: total number of image pixels. Pixel intensities are normalized in the range [0,1]. Included attributes also present busyness [30] (indicating the presence of high frequencies and based on the Sobel edge detector), colorfulness (captures intensity and variations in color using deviations and means of the a and b color channels in the CIELAB color space) [31], lightness (average of the L channel in the CIELAB color space), and complexity [32] (average of the maximum gradient value in the LAB channels). As can be seen in Figure 1, the images presented in the collected dataset are diverse and normally or homogeneously distributed in terms of contrast, lightness, busyness, colorfulness, and complexity. We also checked the distribution of the images’ attributes in each subset, and they show similar results. The results of such a distribution for the RMS contrast attribute is presented in Figure 2, and an example of a relationship between two of the most representative attributes is illustrated in Figure 3. Sample images from the dataset with high and low values for each attribute are shown in Table 1.

### 3.2. Experimental Design

In this study, our objective is to determine the optimal contrast level for natural images that is preferred by individual observers. To achieve this, we utilize an adaptive staircase algorithm and tailor it to our specific requirements. The adaptive staircase algorithm is a widely used [57,58] method to estimate sensory thresholds and adjusts the difficulty level of a task based on the performance of the participant. Adaptive staircase methods are generally considered more efficient and flexible and less reliant on restrictive assumptions than fixed or simple staircase algorithms [57]. While Parameter Estimation by Sequential Testing (PEST) [59] and Quick Estimate of Sensitivity Threshold (QUEST) [60] methods adjust the stimuli intensity based on the observer’s previous correct and incorrect responses, in our case, we the lack ground-truth data and the stimuli threshold that make such adjustment impossible. Although QUEST has a fast convergence time, it relies on many prior assumptions, including knowledge of the stimuli threshold and the use of all previous observer responses to update the prior probability distribution of the threshold, thus limiting its flexibility for our experiment. Instead, we designed our algorithm to be more similar to the PEST procedure, which adjusts the stimulus intensity based on the observer’s previous response, reduces the step size after every response reversal, and increases the step size after four successive choices in the same direction. However, PEST is slower to converge and is sensitive to response biases and sensitive to observers’ correct and incorrect responses, which is not applicable in our case.

To achieve fast convergence while maintaining precision and flexibility, we developed an adaptive algorithm based on a Three-Alternative Forced Choice (3-AFC) procedure. A 3-AFC procedure is more efficient, stable, and precise compared to 2-AFC [61,62,63]. The algorithm used to change the contrast in the image was selected based on the findings of [1,15] and was adopted from the Kadid10K dataset [7]. (The contrast-changing algorithm MATLAB source code can be downloaded from the KADID-10k IQA database webpage: http://database.mmsp-kn.de/kadid-10k-database.html (accessed on 1 November 2021). The name of the function is imcontrastc.m). This algorithm involves adjusting the Sigmoid tonal curve of the RGB image, which influences the luminance and color of the brightness and darkness of different areas. Increasing the contrast makes the bright areas brighter and the dark areas darker, while decreasing the contrast reduces the difference between the brightest and darkest areas.

**Figure 4:**
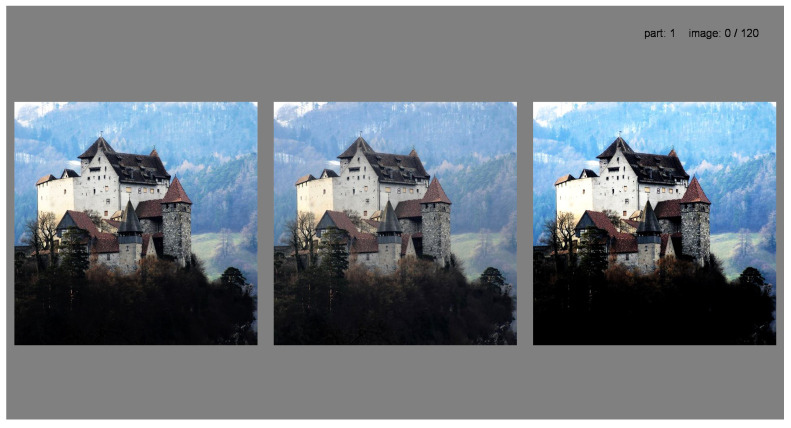
A set of presented images for the first triplet of a trial session to illustrate the initial difference between low-, medium-, and high-contrast images.

**Figure 5:**
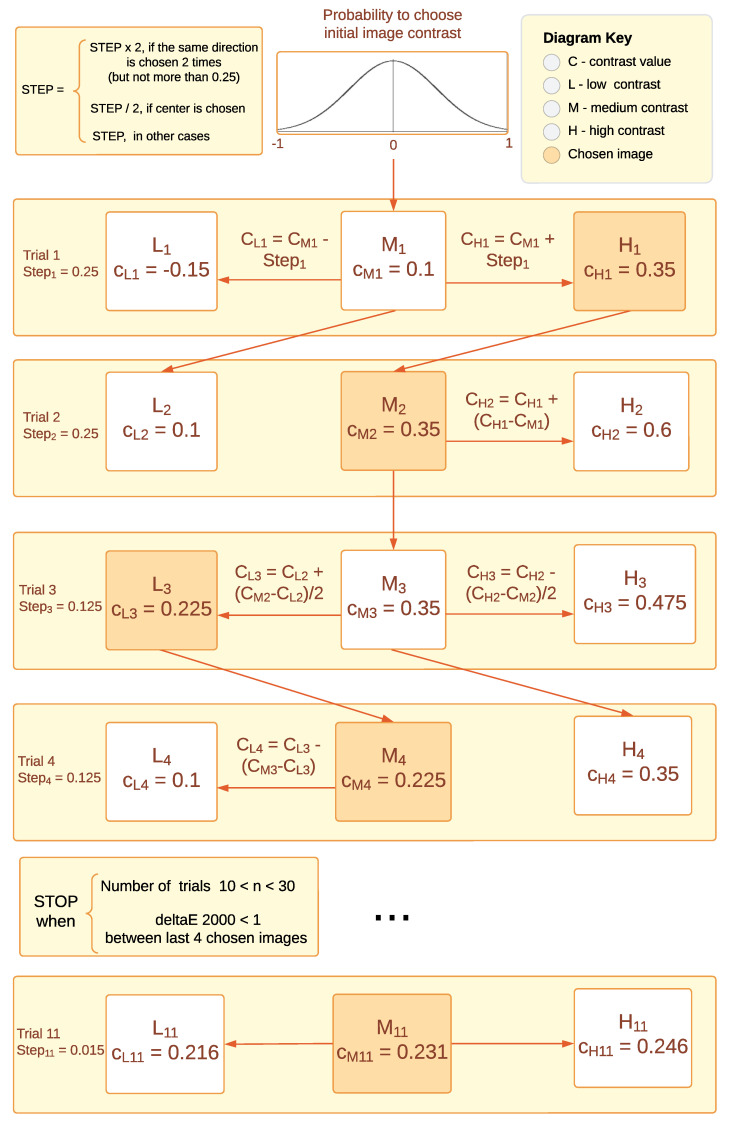
The methodology of our contrast-changing algorithm. Squares correspond to shown images of low, middle, and high contrast, and rows correspond to each trial, for which the chosen image is represented by an orange background. The formulas represent how other images were derived from the chosen one. The step change (contrast difference between images) and stopping rule are also represented.

**Figure 6:**
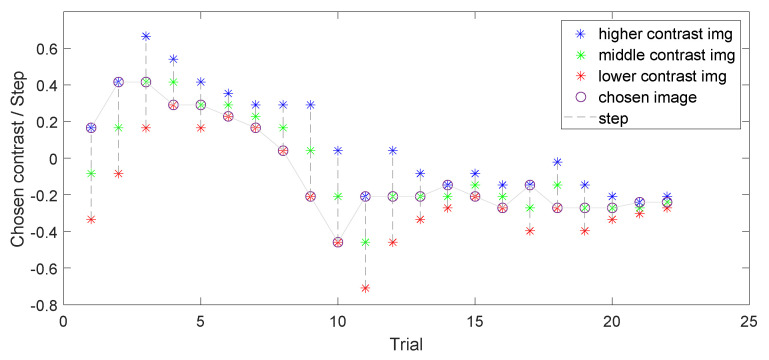
An example of evaluating an image triplet and selecting the preferred contrast by an observer for one image. This is a visualization of the modified adaptive staircase used in this work. Colored asterisks represent displayed images (blue for higher contrast, green for medium contrast, and red for lower contrast). Chosen images are connected with a single line. The step decreases twice after medium contrast is chosen and increases twice after two consecutive choices of higher or lower contrast to avoid a local minimum problem.

At the beginning of the experiment, the observer was presented with three randomly shuffled images of low, medium, and high contrast levels (Figure 4), and each subsequent stimulus level was determined based on the previous response. An example of how the algorithm works is illustrated in Figure 5. The starting level for the medium contrast image was selected from a normally distributed probability range between the minimum and maximum levels of contrast [−1; 1] and had no further impact on algorithm behavior. For the first triplet, the initial difference between low-, medium-, and high-contrast images was set to 0.25. This contrast level was selected to enable easy differentiation between images while avoiding any annoyance forcing the observer to choose the middle contrast level in the first trial. The number of trials ranged from a minimum of 10 to a maximum of 30. During each step, the difference between contrast levels was reduced twice after each choice of the middle contrast, making it more challenging for each subsequent round, and it increased twice after two successive choices of higher or lower contrast. The goal was to reach a level where the observer does not see the difference between images anymore and is satisfied with the contrast level of the three images being displayed. The stopping rule examined the visual difference between the last four chosen images and concluded when Delta-E 2000 [64] remained less than 1, which corresponds to the Just-Noticeable Difference (JND) in color perception as reported by the CIE standard [65]. Although Delta-E 2000 was primarily designed for use with solid colors, it is also used to work with images, and it works well in the design of a stopping rule. An example of the work of this algorithm is shown in Figure 6, wherein 22 trials were done before the stopping rule was enforced. In the first trial, the higher-contrast image was chosen, then in the second trial, the higher-contrast image was chosen; however, the middle-contrast image was selected in the third trial. We can see that when the middle-contrast image was selected, the range for the next trial was reduced to half. Later, for the sixth trial, the lower-contrast image was selected and the range was then further increased.

### 3.3. Experimental Procedure

The experiment was carried out in a controlled environment in a room with ambient illumination of 20 lux at the observer’s location according to ITU [4,66]. An Eizo ColorEdge CG2428 monitor with a resolution of 1920 × 1080 calibrated to sRGB was utilized. To ensure consistency in the experimental conditions, the viewing distance was set to 50 cm: equivalent to a 23-degree viewing angle. In addition, Snellen’s visual acuity and Ishihara’s color blindness tests were administered to each observer.

**Figure 7:**
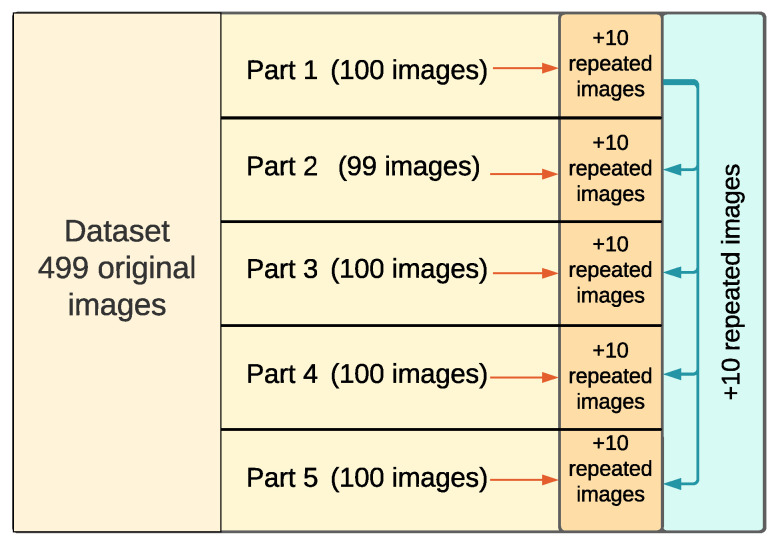
The whole dataset consists of 499 original images split into 5 parts. For consistency checks, 10 images were duplicated inside each part, and 10 images from the first part were repeated in other parts as well.

The entire dataset of 499 images was divided into five subsets, each consisting of 100 images (with the exception of the subset containing 99 images), to allow the observers to finish each subset in an acceptable amount of time. For consistency checks, 20 repeated images were added to each subset (Figure 7). Ten of these images were chosen randomly from the same subset and 10 other images were repeated globally in each subset to check intra-observer variability. In total, 599 (499 unique plus 100 that were repeated in the same or another subset) images were evaluated by each participant. Observers were given the choice to complete as many subsets as they wanted. On average, it took around 1.5 h to complete each subset of images for an observer (not including the rest time). Participants could exit the experiment after any number of completed images and return and continue where they left.

A total of 37 participants (20 male and 17 female) completed at least one subset of images. The participants were 20 to 40 years of age and had normal or corrected normal vision with no color deficiency. The majority of participants (25 people) had a background in either image processing or photography. After the instructions, the observers were presented with a series of three images with varying contrast levels. Participants were instructed to “choose the image you prefer” out of the three images displayed simultaneously. Before the experiment began, observers participated in a brief tutorial session that consisted of completing the entire process for an image. The order of the images was randomized for each participant to minimize any potential order effects. An example showing how the images were presented is illustrated in Figure 4. Out of the 37 observers, 25 finished evaluating all 599 images in the dataset. Observers were given the opportunity to participate in the experiment whenever possible, resulting in the completion time varying from a week to a month for different observers. The data-collection phase of the experiment lasted approximately three months.

## 4. Results and Discussion

### 4.1. Intra-Observer Reliability

To test the reliability of the observers, we included repeated images in our dataset. Each of the five parts of the experiment contained 100 original images (except one part with 99 images) with 10 added images repeated locally in each subset and 10 images repeated across all subsets, which we refer to as locally and globally repeated images, respectively. Therefore, we have 100 contrast values for locally repeated images (10 originals × 5 parts × 2 times) and 50 values for globally repeated images (10 originals × 5 parts) for each observer.

**Figure 8:**
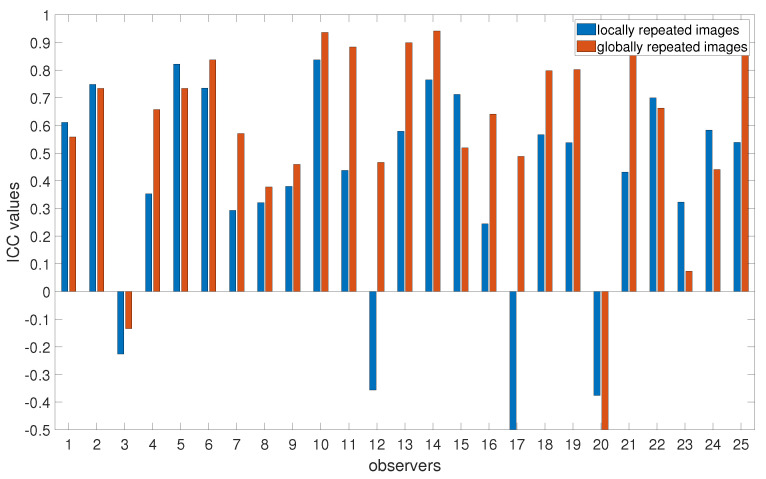
Intraclass Correlation Coefficients (ICCs) for globally and locally repeated images. Higher values indicate higher reliability.

To analyze intra-observer reliability, we use the Intraclass Correlation Coefficient (ICC) [67,68]. ICC is often used to determine correlations within a specific class or cluster of data. It represents both the level of correlation and agreement between the measurements and, therefore, is widely used to measure the reliability of the results. Following the guidelines in [69], we used ICC with 95% confidence intervals based on mean-rating, absolute-agreement, and two-way mixed-effects (A−k):

(2) $$ICC\left(A-k\right)=\frac{MS_{R}-MS_{E}}{MS_{R}+\frac{MS_{C}-MS_{E}}{n}},$$

where $MS_{R}$ corresponds to the Mean Square for the observers (variance between observers), $MS_{E}$ is the Mean Square Error (variance within images, representing random error), $MS_{C}$ is the Mean Square for Cases (variance between images), *n* is the number of images, and *k* is the number of trials (two for local, five for global). ICC is computed by evaluating the variance attributed to observers $MS_{R}$ − $MS_{E}$ in relation to the variance attributed to images $MS_{C}$ − $MS_{E}$ all while taking the sample size *n* into account. The ICC values usually range from −1 to 1, with higher values indicating higher reliability, while negative values indicate poor reliability. The results for the ICC values are presented in Figure 8. The results suggest that Observers 3 and 20 have the poorest reliability for both locally and globally repeated images, while Observers 12 and 17 reveal inconsistencies for locally repeated images. From Figure 8, we can also conclude that ICC values are higher for globally repeated images compared to locally repeated mages. This difference comes from a higher absolute difference in chosen contrast values between the first and second trials for locally repeated images compared to the averaged contrast values for five globally repeated images, which leads to smaller $MS_{E}$ and, in turn, a higher ICC value. Even despite the longer intervals between the evaluations of globally repeated images, the fact that they were assessed multiple times can potentially result in better recall and more consistent responses from observers.

In addition, we conducted an analysis of various indicators to ensure consistency among observers, including Cohen’s kappa [70], mean squared error, standard deviation, and standard error of the mean. To adapt Cohen’s kappa to our case, we discretized our continuous data, ranging from −1 to 1, into 20 categories with a step size of 0.1. This allowed us to assess the consistency between the first and second choices for locally repeated images, while a generalized formula [71] was used to estimate the choices made for globally repeated images. Cohen’s kappa consistency results revealed poor reliability for the same four observers mentioned before as well as for Observer 24. The highest values of mean squared error, standard deviation, and standard error of the mean were detected for the same Observers 3, 12, 17, 20, and 24, which confirms the poor reliability of their results.

**Figure 9:**
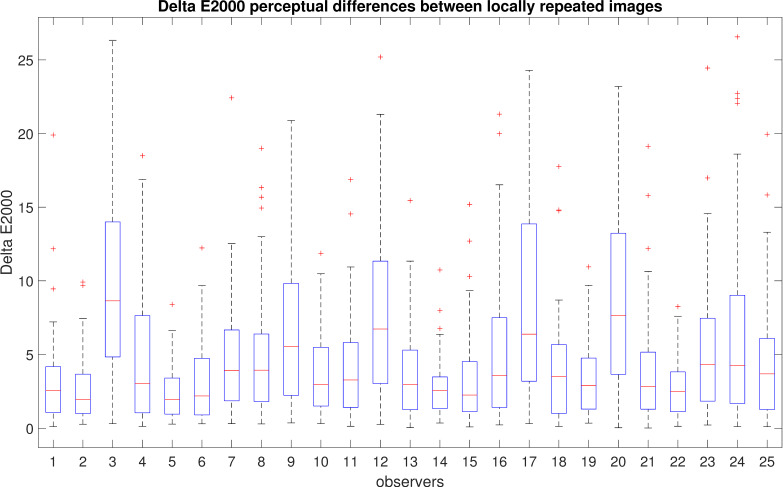
Delta-E 2000 for locally repeated images. Blue boxes indicate the range of majority values between the 25th and 75th percentiles, red lines indicate median values, whiskers extend to the furthest data points, while outliers are indicated by red crosses.

However, comparing only the absolute values of the chosen contrast does not provide insight into the actual differences between the images. For example, how many images with contrast values of −0.2 and −0.3 are perceptually different? To address this, we utilized the Delta-E 2000 formula to evaluate visual differences. We chose Delta-E 2000 because of its simple conversion to perceptual differences. The correspondence between the Delta-E 2000 values and perceptual differences is indicated in Table 2 [72]. Delta-E 2000 differences for pairs of locally repeated images chosen by each observer are presented in Figure 9. We can see that all observers have a mean difference below 10, which means that the difference is perceptible at a glance (Table 2). There exist outliers that have larger color differences (larger than 10) for some observers. There can be multiple reasons for this, such as observers shifting their regions of interest and thereby paying attention to different regions when making their selections. It should also be noted that many observers have very consistent responses, with the interquartile range being below 5 Delta-E 2000.

We also examined the average time taken by observers to choose an image from a set of three. The average decision time for all observers was approximately 1 s. However, Observers 3, 20, and 24 had significantly faster average decision times, which was most likely not enough to make a conscious decision. As a result, we excluded observers 3, 20, and 24 from any further analysis due to their poor reliability and their short time spent on decision making.

### 4.2. Personal Contrast Preferences

**Figure 10:**
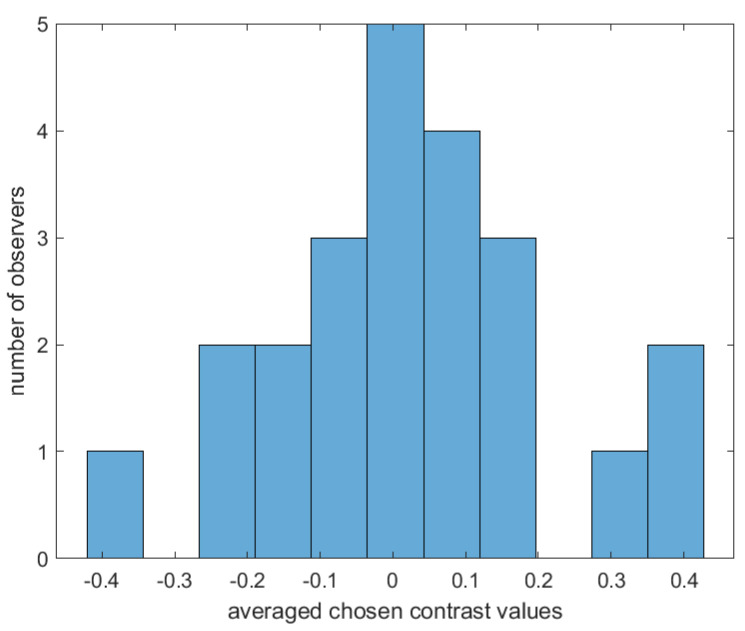
Distribution of preferred contrast level values among observers. The values were averaged for each observer across all 499 images.

**Figure 11:**
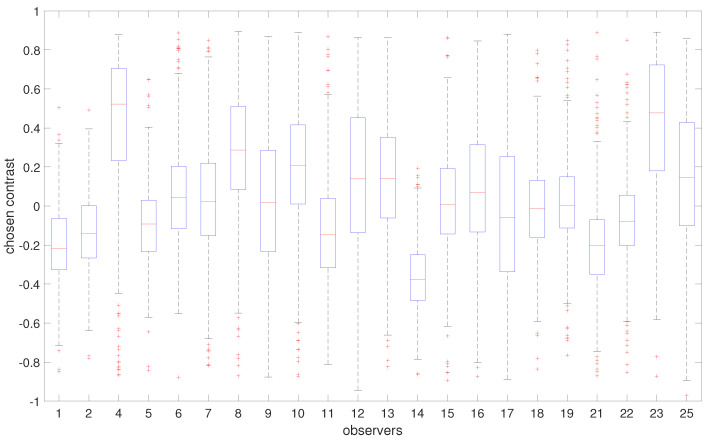
Summary of observers’ preferred contrast. The boxplots show the deviation in observers’ preferences. Some observers have stronger preferences for particular contrast ranges, while the preferences of others vary across different images. Blue boxes indicate the range of majority values between the 25th and 75th percentiles, red lines indicate median values, whiskers extend to the furthest data points, while outliers are indicated by red crosses.

**Figure 12:**
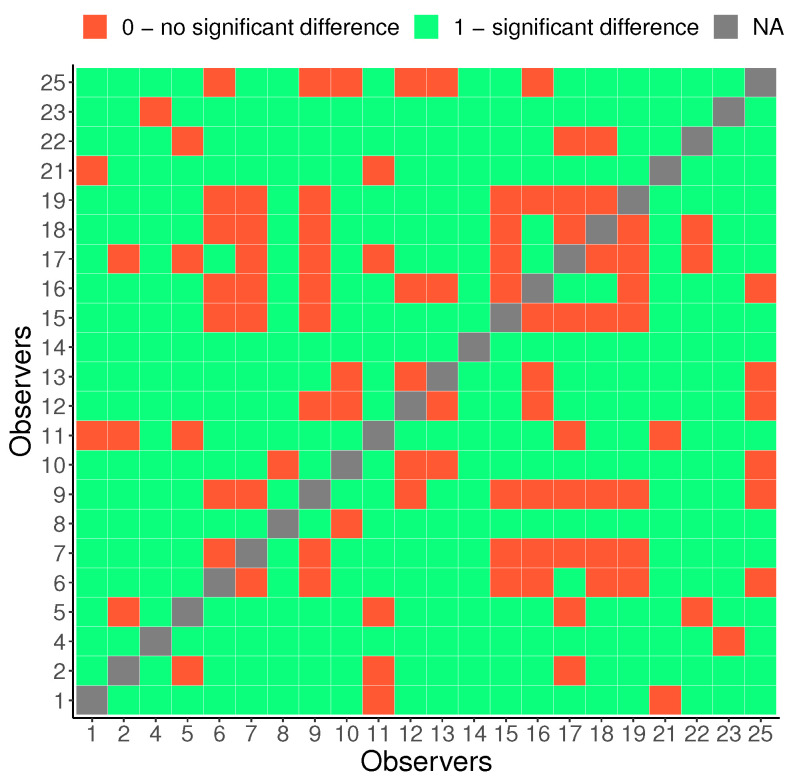
Consistent differences between observers: a sign test with Bonferroni correction.

Our aim is to investigate whether there are variations in contrast preferences among individual observers. Figure 10 shows the distribution of the chosen contrast level values in all images averaged for each observer. Please note that 0 corresponds to an image with slightly higher contrast (0.2 of the original image contrast), which is accounted for in our analysis. Therefore, when we mention 0, we are referring to the “original” image contrast. Although a majority of observers tend to prefer images with contrast levels similar to those of the original image, there is evidence of some disagreement among them. Figure 11 gives more insight into the actual distribution of preferred contrast levels in all images for each observer. However, to determine whether there are significant differences between observers, we performed a sign test with Bonferroni correction [73]. The findings are presented through a confusion matrix (Figure 12), where red indicates no significant differences and green represents the differences between each pair of observers. Through this analysis, we can see that some observers have consistently significantly different responses, while others do not show any such patterns in their responses.

**Figure 13:**
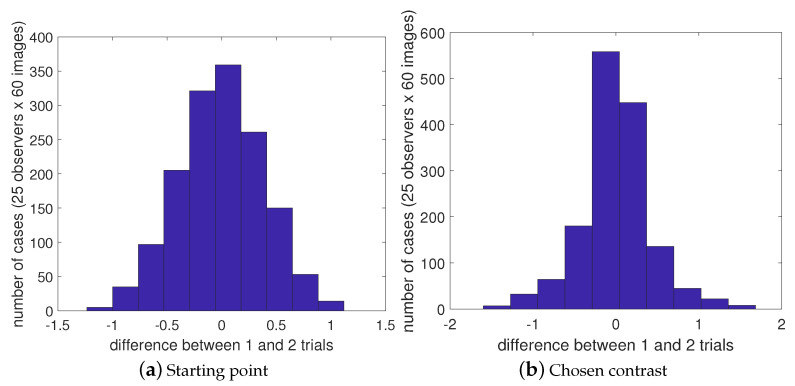
Distribution of the differences in starting points (**a**) and chosen contrast (**b**) between first and second trials for 60 locally repeated images for all observers.

We performed an additional test to ensure a normal distribution for the starting point and chosen contrast in cases of locally repeated images. We examined the differences in starting points and selected contrasts between the first and second trials when evaluating locally repeated images. The differences (Figure 13) were normally distributed around zero, indicating that there was no significant bias or skewness in the randomization of starting points or in the judgments made by the observers.

**Figure 14:**
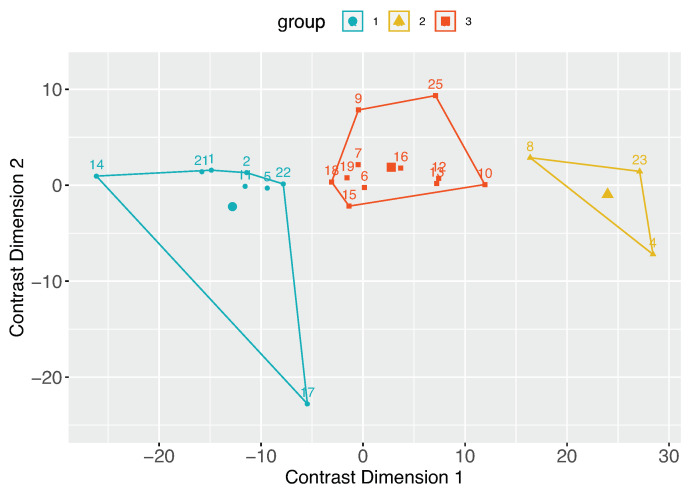
Observers clustered based on their shared contrast preferences. From now on, these are referred to as Group 1, Group 2, and Group 3. Numbers in the figure represent observer IDs.

**Figure 15:**
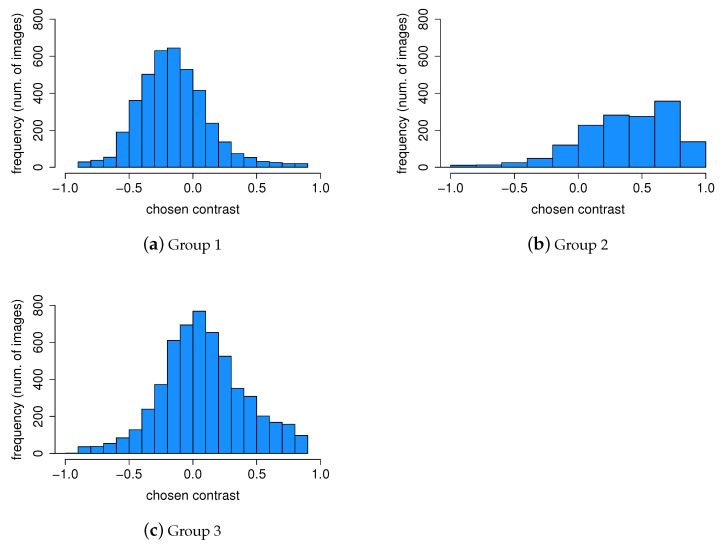
Contrast preference distribution within groups. Group 1 (**a**) prefers lower contrast, Group 2 (**b**) strongly prefers high-contrast images, while Group 3 (**c**) prefers images with slightly higher and natural contrast.

**Figure 16:**
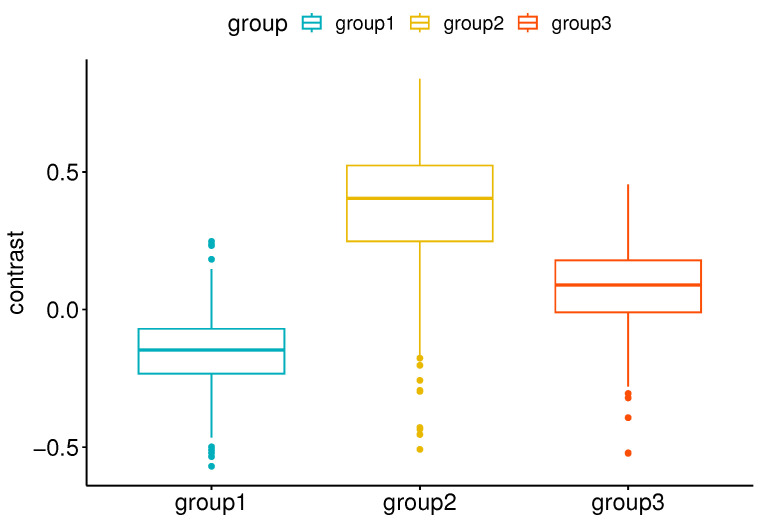
Boxplots show the distributions of contrast preferences within each group.

An important part of our work is to study if there are distinct groups of observers with similar contrast preferences. To achieve this, we employ the k-means clustering algorithm. The resulting groups are visualized in Figure 14. Given that we have a single type of data used for clustering, the axes Contrast Dimension 1 and 2 in Figure 14 represent the two most informative derived dimensions (principal components) within the transformed space, which capture the most substantial variation in the data. By analyzing the distributions within these groups (Figure 15), we can identify distinct groups of observers: those who seem to prefer images with lower contrast, for whom the histogram is shifted towards the left (Group 1, Figure 15a); those who seem to prefer images with slightly higher contrast or contrast-rich images, for whom the histogram is shifted right (Group 2, Figure 15b); and those who seem to prefer images with contrast levels similar to the original image, for whom the histogram is centered around 0 (Group 3, Figure 15c). The cluster separation is determined along the Contrast Dimension 1 axis, which represents the chosen contrast values. Observers distributed along the *x*-axis correspond to those in Figure 16. For example, Observers 14, 21, 1, 2, 11, 5, 22, and 17 have median preferred contrast values below zero according to Figure 16, while Observers 8, 23, and 4 have the highest median preferred contrasts.

In Figure 16, we can see the differences in the distributions of preferred contrast values represented in the boxplots for each group. To determine if the differences between the groups are significant, we can use parametric or nonparametric tests. First, we checked if our data are normally distributed. For this, we ran the Shapiro–Wilk [74] and Kolmogorov–Smirnov [75] tests. Both tests reported significantly small *p*-values, indicating that the data are not normally distributed. Therefore, we chose the Kruskal–Wallis test [76] to check if there are significant differences between the three groups and the Wilcoxon signed-rank test on paired samples [77] to check the significance between each pair of groups. The Wilcoxon test assumes that the differences between paired samples are symmetrically distributed around the median, and this assumption is satisfied in our case. The results of the tests are shown in Table 3. Both tests show statistically significant differences between the means of the groups, with *p*-values close to zero.

**Figure 17:**
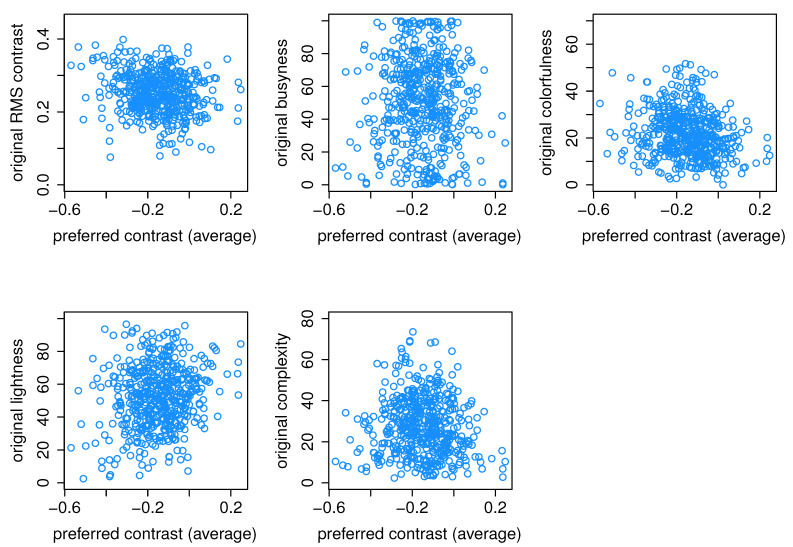
Scatter plot of original image attributes vs. preferred image contrast for Group 1.

We also explored the relationship between the image attributes (Figure 1) and the preferred contrast levels within each group. This analysis is aimed to determine whether the appearance of an image can provide valuable insights into contrast preferences for the observers. Figure 17 displays, as an example, a scatter plot between five image attributes: original RMS contrast, busyness, lightness, colorfulness, complexity, and preferred contrast levels averaged for observers of Group 1, but no significant relationship was found. Although there is a slight tendency for images with lower complexity and higher lightness to be preferred with higher contrast, it is important to note that these relationships are not statistically significant. Similarly, we checked Groups 2 and 3, and no significant relationships were found either.

**Figure 18:**
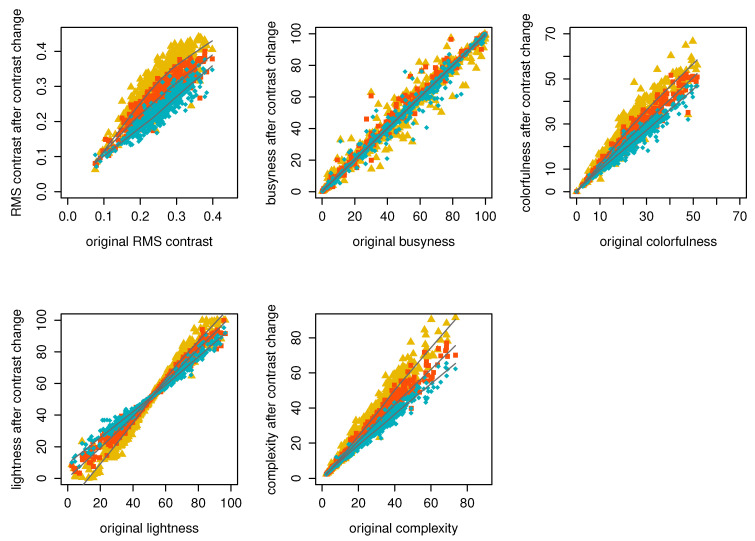
Scatter plots of chosen image attributes for Group 1 (yellow), Group 2 (orange), and Group 3 (blue) against original image attributes. Gray median lines are provided to approximate the distribution patterns and mitigate occlusions.

To see if there are differences between images chosen by the three groups, we also plotted each of the attributes against the original image attributes. Figure 18 represents the distribution of each of the 499 image attributes corresponding to an averaged image chosen by the observers of each of the three groups (i.e., preferred contrast value was averaged for each image within each group). Median lines help to see approximate distributions and mitigate occlusions. From the plots, we can see the changes in each attribute. RMS contrast shows that the original contrast is lower for Group 1, higher for Group 2, and slightly higher for Group 3, which supports the results and Figure 15 and Figure 16. Busyness, which is based on the Sobel edge detector, is not affected by contrast change. Colorfulness increases with increasing contrast, as does complexity; these are slightly increased for Groups 1 and 2. Because complexity is related to the maximum gradient values in the L, A, and B color channels, and the gradient values in the L color channel increase with higher contrast, this supports our findings. Colorfulness is related to the mean and deviation of the a and b channels; it also increases with higher contrast. For lightness, dark images become darker and bright images become brighter, which happens for Group 2 with increasing contrast, while the opposite happens for Group 3, for whom images with extreme lightness levels are flattened towards the middle while middle lightness level 50 stays the same, which also supports our findings.

In this study, we did not include the original image contrast as a weighting factor in our calculations because the primary focus was on understanding the relative individual differences between observers when evaluating the same images. However, it would be an interesting addition to examine images with similar original contrast levels and make comparisons. Due to the limited number of observers, we did not investigate the influence of the background and cultural differences of the observers in this work. This question will be addressed in a forthcoming study.

This research is the first step in understanding individual differences in image quality preferences. By identifying and modeling these individual variations, we can use this knowledge to optimize image enhancement models. The applications of such optimization extend to virtual reality, augmented reality, and the entertainment industry. The database presented here, along with the accompanying results, is available for modeling observers’ preferences for image contrast. It can be downloaded from www.colourlab.no. This addresses the existing gap in having a database with data from each individual assessment. Researchers can use the ground-truth data to test and improve the performance of existing and future contrast-based IQMs and tailor them to individual groups or observers if desired.

## 5. Conclusions

In this study, we investigated individual contrast preferences and identified statistically significant groups of observers who demonstrated preferences for low-, natural-, and high-contrast images. The results show the existence of individual differences among observers for contrast preferences, which should be taken into account in IQA, image enhancement, and other related fields. A total of 499 contrast preference values from 22 observers were collected for analysis. To collect these preferences, we used a 3-AFC procedure combined with a modified adaptive staircase algorithm, which ensured fast convergence and maintained high precision. This database is available online to use for further research on personalized image quality assessment.

Further research will be carried out to investigate how we can predict observers’ contrast preferences. The dataset presented in this study serves as a valuable resource for conducting such analyses by offering a solid foundation for future investigations. Our findings support the presence of individual preferences in the level of contrast of each image. This research can also be expanded to explore other image attributes, including saturation, lightness, sharpness, and more. By developing the ability to predict these features, we can make significant contributions to the field of personalized IQA and image enhancement.

## Figures and Tables

**Table 2:** Delta-E 2000 values for perception correspondence [65].

Delta-E 2000	Perception
≤1.0	Not perceptible by human eyes.
1–2	Perceptible through close observation.
2–10	Perceptible at a glance.
11–49	Colors are more similar than opposite.
100	Colors are exact opposite.

**Table 3:** Wilcoxon signed-rank test on paired samples. Output from R. **** presents statistically significant difference between groups.

	Group	Group	n1	n2	*p*	*p*.adj	*p*.adj.signif
1	Group 1	Group 2	499	499	<0.01	<0.01	****
2	Group 1	Group 3	499	499	<0.01	<0.01	****
3	Group 2	Group 3	499	499	<0.01	<0.01	****

## Data Availability

The database with individual contrast preferences and additional information on other image attributes reported in this work can be found at www.colourlab.no from 1 February 2024.

## Abbreviations

The following abbreviations are used in this manuscript:

IQA	Image Quality Assessment
IQM	Image Quality Metric
MOS	Mean Opinion Score
3-AFC	Three-Alternative Forced Choice
JND	Just-Noticeable Difference
ICC	Intraclass Correlation Coefficient

## References

[1] Cherepkova O., Amirshahi S.A., Pedersen M. Analyzing the Variability of Subjective Image Quality Ratings for Different Distortions. Proceedings of the 2022 Eleventh International Conference on Image Processing Theory, Tools and Applications (IPTA).

[2] Ren J., Shen X., Lin Z., Mech R., Foran D.J. Personalized image aesthetics. Proceedings of the IEEE International Conference on Computer Vision.

[3] ITU (2017). Vocabulary for Performance, Quality of Service and Quality of Experience.

[4] CIE (2003). Guidelines for the evaluation of gamut mapping algorithms. Publ.-Comm. Int. Eclair. Cie.

[5] ITU (2016). Methods for the Subjective Assessment of Video Quality Audio Quality and Audiovisual Quality of Internet Video and Distribution Quality Television in any Environment.

[6] Field G.G. (1999). Test image design guidelines for color quality evaluation. Color and Imaging Conference.

[7] Lin H., Hosu V., Saupe D. KADID-10k: A large-scale artificially distorted IQA database. Proceedings of the 2019 Eleventh International Conference on Quality of Multimedia Experience (QoMEX).

[8] Ghadiyaram D., Bovik A.C. (2015). Massive online crowdsourced study of subjective and objective picture quality. IEEE Trans. Image Process..

[9] Ponomarenko N., Jin L., Ieremeiev O., Lukin V., Egiazarian K., Astola J., Vozel B., Chehdi K., Carli M., Battisti F. (2015). Image database TID2013: Peculiarities, results and perspectives. Signal Process. Image Commun..

[10] Partos T.R., Cropper S.J., Rawlings D. (2016). You don’t see what I see: Individual differences in the perception of meaning from visual stimuli. PLoS ONE.

[11] Owsley C., Sekuler R., Siemsen D. (1983). Contrast sensitivity throughout adulthood. Vis. Res..

[12] Cornsweet T. (2012). Visual Perception.

[13] Roufs J., Goossens I. The effect of gamma on perceived image quality. Proceedings of the Conference Record of the 1988 International Display Research Conference.

[14] Beghdadi A., Qureshi M.A., Amirshahi S.A., Chetouani A., Pedersen M. (2020). A Critical Analysis on Perceptual Contrast and Its Use in Visual Information Analysis and Processing. IEEE Access.

[15] Cherepkova O., Amirshahi S.A., Pedersen M. (2022). Analysis of individual quality scores of different image distortions. Color and Imaging Conference (CIC).

[16] Azimian S., Torkamani-Azar F., Amirshahi S.A. (2021). How good is too good? A subjective study on over enhancement of images. Color and Imaging Conference (CIC).

[17] Azimian S., Amirshahi S.A., Azar F.T. (2023). Preventing Over-Enhancement Using Modified ICSO Algorithm. IEEE Access.

[18] Roufs J.A., Koselka V.J., van Tongeren A.A. Global brightness contrast and the effect on perceptual image quality. Proceedings of the Human Vision, Visual Processing, and Digital Display V.

[19] Varga D. (2021). No-reference image quality assessment with global statistical features. J. Imaging.

[20] Chen S.D. (2012). A new image quality measure for assessment of histogram equalization-based contrast enhancement techniques. Digit. Signal Process..

[21] Ziaei Nafchi H., Cheriet M. (2018). Efficient No-Reference Quality Assessment and Classification Model for Contrast Distorted Images. IEEE Trans. Broadcast..

[22] Liu Y., Li X. (2020). No-Reference Quality Assessment for Contrast-Distorted Images. IEEE Access.

[23] Fang Y., Ma K., Wang Z., Lin W., Fang Z., Zhai G. (2015). No-Reference Quality Assessment of Contrast-Distorted Images Based on Natural Scene Statistics. IEEE Signal Process. Lett..

[24] Michelson A. (1927). Studies in Optics.

[25] Attneave F. (1954). Some informational aspects of visual perception. Psychol. Rev..

[26] Marr D. (2010). Vision: A Computational Investigation into the Human Representation and Processing of Visual Information.

[27] Haralick R.M., Shapiro L.G. (1985). Image segmentation techniques. Comput. Vision, Graph. Image Process..

[28] Calabria A.J., Fairchild M.D. (2003). Perceived image contrast and observer preference II. Empirical modeling of perceived image contrast and observer preference data. J. Imaging Sci. Technol..

[29] Kadyrova A., Pedersen M., Ahmad B., Mandal D.J., Nguyen M., Zimmermann P. (2022). Image enhancement dataset for evaluation of image quality metrics. IS&T International Symposium on Electronic Imaging Science and Technology.

[30] Orfanidou M., Triantaphillidou S., Allen E. (2008). Predicting image quality using a modular image difference model. Proceedings of the Image Quality and System Performance V.

[31] Hasler D., Suesstrunk S.E. (2003). Measuring colorfulness in natural images. Proceedings of the Human Vision and Electronic Imaging VIII.

[32] Redies C., Amirshahi S.A., Koch M., Denzler J. (2012). PHOG-derived aesthetic measures applied to color photographs of artworks, natural scenes and objects. Proceedings of the Computer Vision–ECCV 2012. Workshops and Demonstrations.

[33] Amirshahi S.A. (2015). Aesthetic Quality Assessment of Paintings. Ph.D. Thesis.

[34] Amirshahi S.A., Hayn-Leichsenring G.U., Denzler J., Redies C. (2015). Jenaesthetics subjective dataset: Analyzing paintings by subjective scores. Lect. Notes Comput. Sci..

[35] Li J., Datta R., Joshi D., Wang J. (2006). Studying aesthetics in photographic images using a computational approach. Lect. Notes Comput. Sci..

[36] Ke Y., Tang X., Jing F. The design of high-level features for photo quality assessment. Proceedings of the 2006 IEEE Computer Society Conference on Computer Vision and Pattern Recognition (CVPR 2006).

[37] Dhar S., Ordonez V., Berg T.L. High level describable attributes for predicting aesthetics and interestingness. Proceedings of the 2011 IEEE Computer Society Conference on Computer Vision and Pattern Recognition (CVPR 2011).

[38] Segalin C., Perina A., Cristani M., Vinciarelli A. (2016). The pictures we like are our image: Continuous mapping of favorite pictures into self-assessed and attributed personality traits. IEEE Trans. Affect. Comput..

[39] Lovato P., Bicego M., Segalin C., Perina A., Sebe N., Cristani M. (2014). Faved! biometrics: Tell me which image you like and I’ll tell you who you are. IEEE Trans. Inf. Forensics Secur..

[40] Li L., Zhu H., Zhao S., Ding G., Lin W. (2020). Personality-assisted multi-task learning for generic and personalized image aesthetics assessment. IEEE Trans. Image Process..

[41] Bhandari U., Chang K., Neben T. (2019). Understanding the impact of perceived visual aesthetics on user evaluations: An emotional perspective. Inf. Manag..

[42] Yang Y., Xu L., Li L., Qie N., Li Y., Zhang P., Guo Y. Personalized image aesthetics assessment with rich attributes. Proceedings of the IEEE/CVF Conference on Computer Vision and Pattern Recognition.

[43] Zhu H., Zhou Y., Shao Z., Du W., Wang G., Li Q. (2022). Personalized Image Aesthetics Assessment via Multi-Attribute Interactive Reasoning. Mathematics.

[44] Park K., Hong S., Baek M., Han B. Personalized image aesthetic quality assessment by joint regression and ranking. Proceedings of the 2017 IEEE Winter Conference on Applications of Computer Vision (WACV).

[45] Zhu H., Li L., Wu J., Zhao S., Ding G., Shi G. (2020). Personalized image aesthetics assessment via meta-learning with bilevel gradient optimization. IEEE Trans. Cybern..

[46] Lv P., Fan J., Nie X., Dong W., Jiang X., Zhou B., Xu M., Xu C. (2021). User-guided personalized image aesthetic assessment based on deep reinforcement learning. IEEE Trans. Multimed..

[47] Cui C., Yang W., Shi C., Wang M., Nie X., Yin Y. (2020). Personalized image quality assessment with social-sensed aesthetic preference. Inf. Sci..

[48] Kim H.U., Koh Y.J., Kim C.S. (2020). PieNet: Personalized image enhancement network. Proceedings of the Computer Vision–ECCV 2020: 16th European Conference.

[49] Kang S.B., Kapoor A., Lischinski D. Personalization of image enhancement. Proceedings of the 2010 IEEE Computer Society Conference on Computer Vision and Pattern Recognition.

[50] Bianco S., Cusano C., Piccoli F., Schettini R. (2020). Personalized image enhancement using neural spline color transforms. IEEE Trans. Image Process..

[51] Caicedo J.C., Kapoor A., Kang S.B. Collaborative personalization of image enhancement. Proceedings of the CVPR 2011.

[52] Gigilashvili D., Thomas J.B., Pedersen M., Hardeberg J.Y. Perceived glossiness: Beyond surface properties. Proceedings of the Color and Imaging Conference. Society for Imaging Science and Technology.

[53] Engelke U., Pitrey Y., Le Callet P. Towards an inter-observer analysis framework for multimedia quality assessment. Proceedings of the 2011 Third International Workshop on Quality of Multimedia Experience.

[54] Zhang B., Allebach J.P., Pizlo Z. (2005). An investigation of perceived sharpness and sharpness metrics. Proceedings of the Image Quality and System Performance II.

[55] Pixabay. https://pixabay.com.

[56] Achanta R., Hemami S., Estrada F., Susstrunk S. Frequency-tuned salient region detection. Proceedings of the 2009 IEEE Conference on Computer Vision and Pattern Recognition.

[57] Leek M.R. (2001). Adaptive procedures in psychophysical research. Percept. Psychophys..

[58] Lu Z.L., Dosher B. (2013). Adaptive Psychophysical Procedures. Visual Psychophysics: From Laboratory to Theory.

[59] Hall J.L. (1981). Hybrid adaptive procedure for estimation of psychometric functions. J. Acoust. Soc. Am..

[60] Watson A.B., Pelli D.G. (1983). QUEST: A Bayesian adaptive psychometric method. Percept. Psychophys..

[61] Mantiuk R.K., Tomaszewska A., Mantiuk R. (2012). Comparison of four subjective methods for image quality assessment. Computer Graphics Forum.

[62] Shelton B., Scarrow I. (1984). Two-alternative versus three-alternative procedures for threshold estimation. Percept. Psychophys..

[63] Schlauch R.S., Rose R.M. (1990). Two-, three-, and four-interval forced-choice staircase procedures: Estimator bias and efficiency. J. Acoust. Soc. Am..

[64] Sharma G., Wu W., Dalal E.N. (2005). The CIEDE2000 color-difference formula: Implementation notes, supplementary test data, and mathematical observations. Color Res. Appl..

[65] Karma I.G.M. (2020). Determination and Measurement of Color Dissimilarity. Int. J. Eng. Emerg. Technol..

[66] Bt Recommendation ITU-R (2002). Methodology for the Subjective Assessment of the Quality of Television Pictures.

[67] McGraw K.O., Wong S.P. (1996). Forming inferences about some intraclass correlation coefficients. Psychol. Methods.

[68] Salarian A. (2023). Intraclass Correlation Coefficient (ICC). https://www.mathworks.com/matlabcentral/fileexchange/22099-intraclass-correlation-coefficient-icc.

[69] Koo T.K., Li M.Y. (2016). A guideline of selecting and reporting intraclass correlation coefficients for reliability research. J. Chiropr. Med..

[70] Landis J.R., Koch G.G. (1977). The measurement of observer agreement for categorical data. Biometrics.

[71] Girard J.M. MATLAB Functions for Computing Inter-Observer Reliability. 2016–2021. https://www.mathworks.com/matlabcentral/fileexchange/64602-matlab-functions-for-computing-inter-observer-reliability.

[72] Schuessler Z. (2016). Delta E 101. http://zschuessler.github.io/DeltaE/learn/.

[73] Lehmann E.L., D’Abrera H.J. (1975). Nonparametrics: Statistical Methods Based on Ranks.

[74] Shapiro S.S., Wilk M.B. (1965). An analysis of variance test for normality (complete samples). Biometrika.

[75] Smirnov N.V. (1939). Estimate of deviation between empirical distribution functions in two independent samples. Bull. Mosc. Univ..

[76] Kruskal W.H., Wallis W.A. (1952). Use of ranks in one-criterion variance analysis. J. Am. Stat. Assoc..

[77] Litchfield J.J., Wilcoxon F. (1949). A simplified method of evaluating dose-effect experiments. J. Pharmacol. Exp. Ther..

